# Supplementary material for: Long-read sequencing unveils IGH-DUX4 translocation into the silenced IGH allele in B-cell acute lymphoblastic leukemia
Source: Nat Commun. 2019 Jun 26;10:2789. doi: 10.1038/s41467-019-10637-8 (PMC6594946; doi:10.1038/s41467-019-10637-8)
Supplement: Supplementary file 1 — Supplementary Information [file 41467_2019_10637_MOESM1_ESM.pdf]

Long-read sequencing unveils *IGH-DUX4* translocation into the silenced *IGH* allele in B-cell acute lymphoblastic leukemia

Tian *et al.*

Supplementary Information

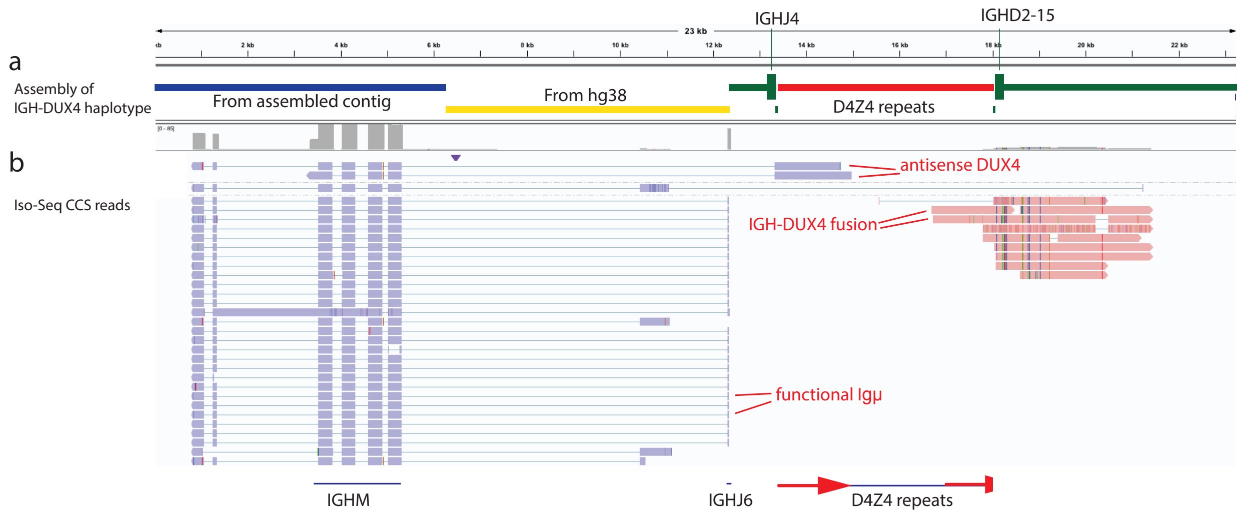

Supplementary Figure 1 Full-length transcripts near *IGH-DUX4* translocation region.

(a) Manually assembled DNA sequence near *IGH-DUX4* translocation region. The sequences from Yasuda et al.[1] are marked in green. (b) Mapping result of Iso-Seq CCS reads to the assembled DNA sequence. The majority of the reads were in-frame coding for functional Igμ. Two *IGH-DUX4* fusion transcripts were identified, and the sequence of the shorter was supported by the 3’ RACE experiment by Yasuda et al.[1] Antisense *DUX4* transcript containing the *IGHM* exons was also identified.

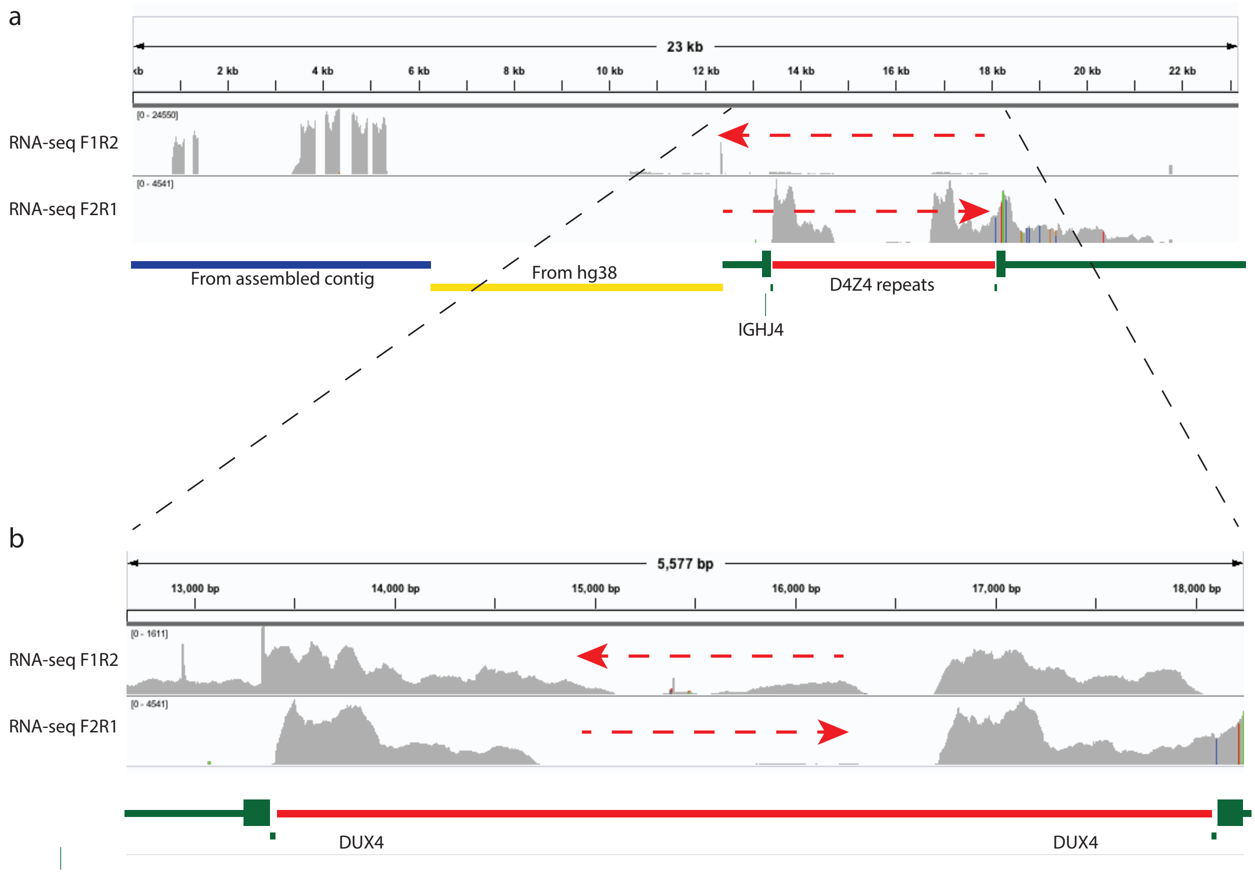

Supplementary Figure 2 Bidirectional transcription of rearranged *DUX4* in Nalm6.

(a) Mapping result of stranded RNA-seq reads to the assembled DNA sequence near *IGH-DUX4* translocation in Nalm6. Reverse and forward reads were shown in two tracks and marked with red arrows. (b) Zoomed in view at the rearranged *DUX4* region. Both sense and antisense *DUX4* were transcribed.

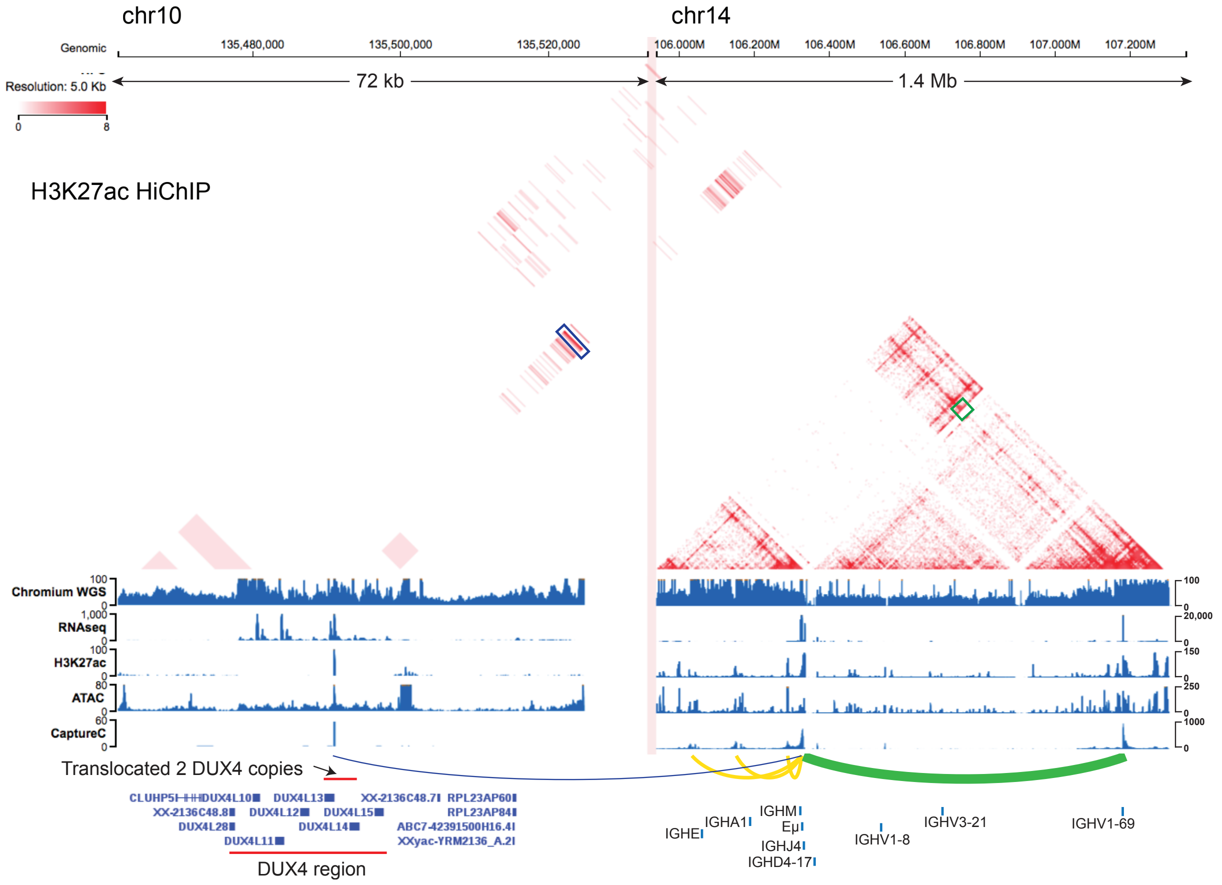

Supplementary Figure 3 Chromatin interaction plot from H3K27ac HiChIP data at the *DUX4* region in chr10 and the *IGH* region.

Data were visualized in ProteinPaint[2]. The Eμ-*DUX4* interaction and Eμ-Igμ promoter interaction are marked as a blue and green box, respectively. The colored arcs show Capture-C identified interaction regions with Eμ, described in Fig. 3a. Different scales are used at *DUX4* and *IGH* region for RNA-seq, H3K27ac, ATAC-seq and Capture-C.

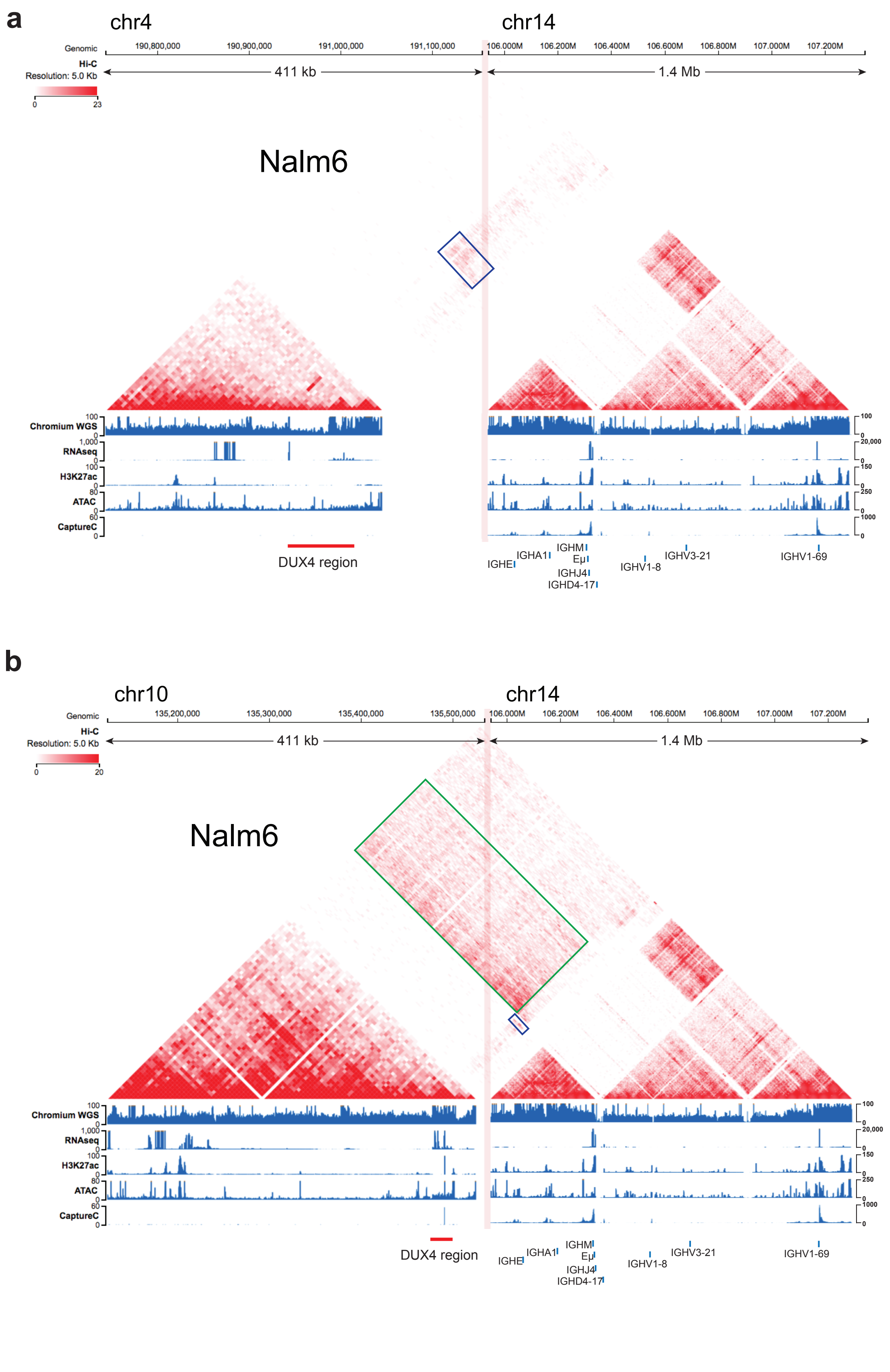
a
chr4
chr14
Genomic
Hi-C
Resolution: 5.0 Kb
190,800,000
190,900,000
191,000,000
191,100,000
106.000M
106.200M
106.400M
106.600M
106.800M
107.000M
107.200M
411 kb
1.4 Mb
Nalm6
Chromium WGS
RNAseq
H3K27ac
ATAC
CaptureC
DUX4 region
IGHE
IGHA1
IGHM
Eμ
IGHJ4
IGHD4-17
IGHV1-8
IGHV3-21
IGHV1-69
b
chr10
chr14
Genomic
Hi-C
Resolution: 5.0 Kb
135,200,000
135,300,000
135,400,000
135,500,000
106.000M
106.200M
106.400M
106.600M
106.800M
107.000M
107.200M
411 kb
1.4 Mb
Nalm6
Chromium WGS
RNAseq
H3K27ac
ATAC
CaptureC
DUX4 region
IGHE
IGHA1
IGHM
Eμ
IGHJ4
IGHD4-17
IGHV1-8
IGHV3-21
IGHV1-69

Supplementary Figure 4 Comparison of Hi-C plot between *IGH-DUX4*(chr4) and *IGH-DUX4*(chr10) in Nalm6.

(a) Hi-C plot between chr4 telomere and chr14 *IGH*. (b) Hi-C plot between chr10 telomere and chr14 *IGH*. Because *DUX4* array exists in both chr4 and chr10 telomere, the *IGH-DUX4* interaction shown in both chr4 and chr10 *DUX4* region (blue boxes). The green box shown interaction signal between the left side of chr10 *DUX4* region and *IGH*, which was explained as a reciprocal translocation event described in Methods. Data were visualized in ProteinPaint[2]. Different scales are used at *DUX4* and *IGH* region for RNA-seq, H3K27ac, ATAC-seq and Capture-C.

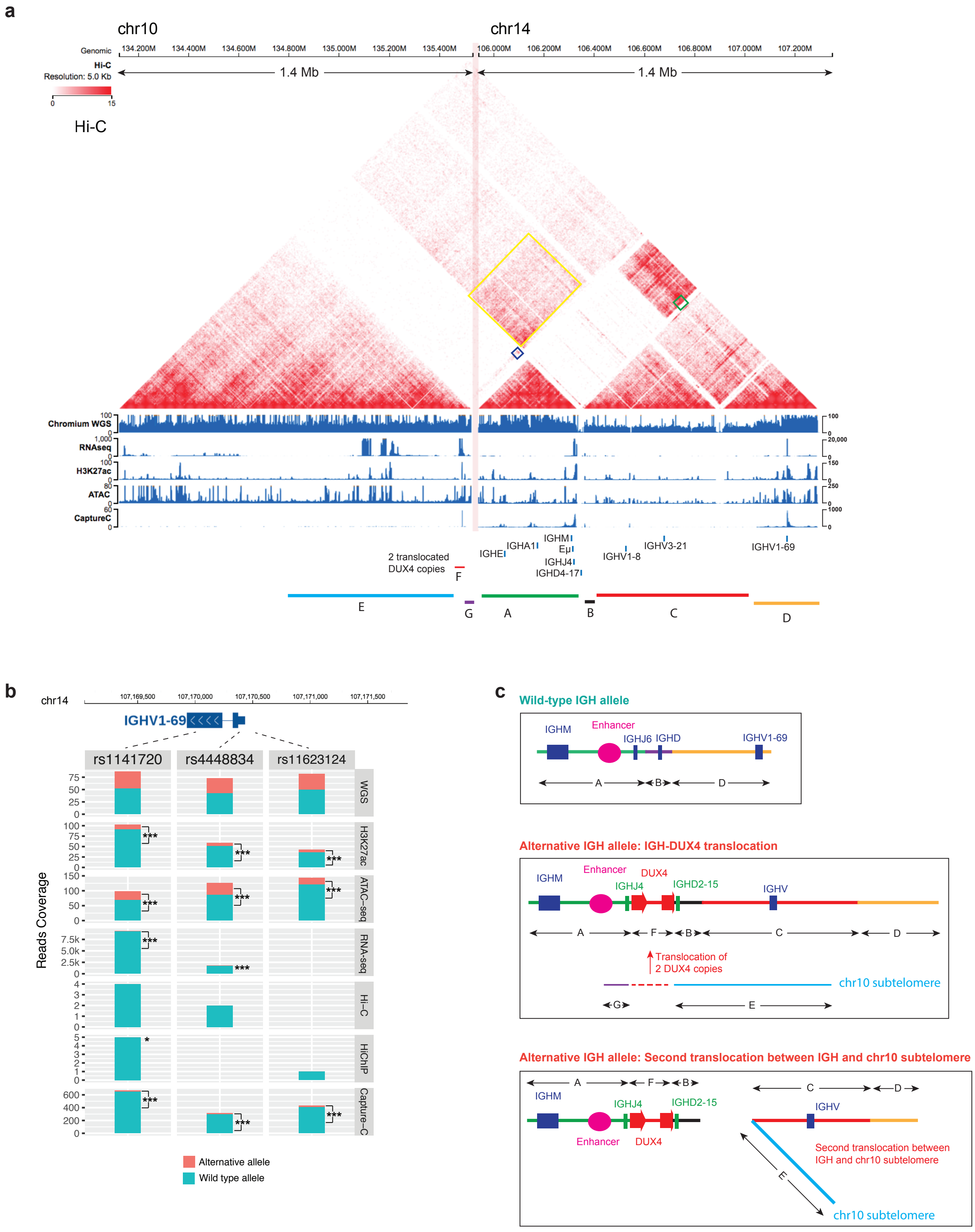
a
chr10
chr14
Genomic
Hi-C
Resolution: 5.0 Kb
1.4 Mb
1.4 Mb
Hi-C
Chromium WGS
RNAseq
H3K27ac
ATAC
CaptureC
2 translocated
DUX4 copies
F
IGHE
IGHA1
IGHM
Eμ
IGHJ4
IGHD4-17
IGHV1-8
IGHV3-21
IGHV1-69
E
G
A
B
C
D
b
chr14
IGHV1-69
rs1141720
rs4448834
rs11623124
WGS
H3K27ac
ATAC–seq
RNA–seq
Hi–C
HIChIP
Capture–C
Reads Coverage
Alternative allele
Wild type allele
c
Wild-type IGH allele
IGHM
Enhancer
IGHJ6
IGHD
IGHV1-69
Alternative IGH allele: IGH-DUX4 translocation
IGHM
Enhancer
DUX4
IGHJ4
IGHD2-15
IGHV
Translocation of
2 DUX4 copies
chr10 subtelomere
Alternative IGH allele: Second translocation between IGH and chr10 subtelomere
IGHM
Enhancer
IGHJ4
DUX4
IGHD2-15
IGHV
Second translocation between
IGH and chr10 subtelomere
chr10 subtelomere

Supplementary Figure 5 A reciprocal translocation between chr10 subtelomere and *IGH* region revealed from Hi-C data.

(a) The Hi-C interaction plot between 1.4 Mb region of chr10 telomere and the *IGH* region. The Eμ-*DUX4* interaction and Eμ-Igμ promoter interaction are marked as a blue and green box, respectively. Yellow box supported the reciprocal translocation between chr10 subtelomere and *IGH* region (Details in Methods). Data were visualized in ProteinPaint[2]. Different scales are used at *DUX4* and *IGH* region for RNA-seq, H3K27ac, ATAC-seq and Capture-C. Regions marked from ‘’A” to “G” with different color were used to illustrator 2 translocation events between chr10 subtelomere and *IGH* region in panel c. (b) Evaluation of allelic imbalance at heterozygous SNPs (rs1141720, rs4448834, rs11623124) near Igμ promoter. Two haplotypes “C-T-C” and “T-C-G” were phased from Chromium WGS at these SNPs. The haplotype “C-T-C” stands for *IGH* wild-type allele because our Iso-Seq long reads for Igμ shown the genotype “C-T” at this exonic region and “A-A” at *IGHM* exonic region. For RNA-seq, only exonic SNPs (rs1141720, rs4448834) are considered. For Hi-C and HiChIP data, only the reads contacted with Eμ were calculated. One-tailed binomial test (expected probability 0.5) was performed: * $p<0.05$, ** $p<0.01$, *** $p<0.001$. (c) A model for the 2 translocation events between chr10 subtelomere and *IGH* region interpreted from Hi-C data. Regions marked from ‘’A” to “G” with different color were consistent with panel a. Details are in Methods.

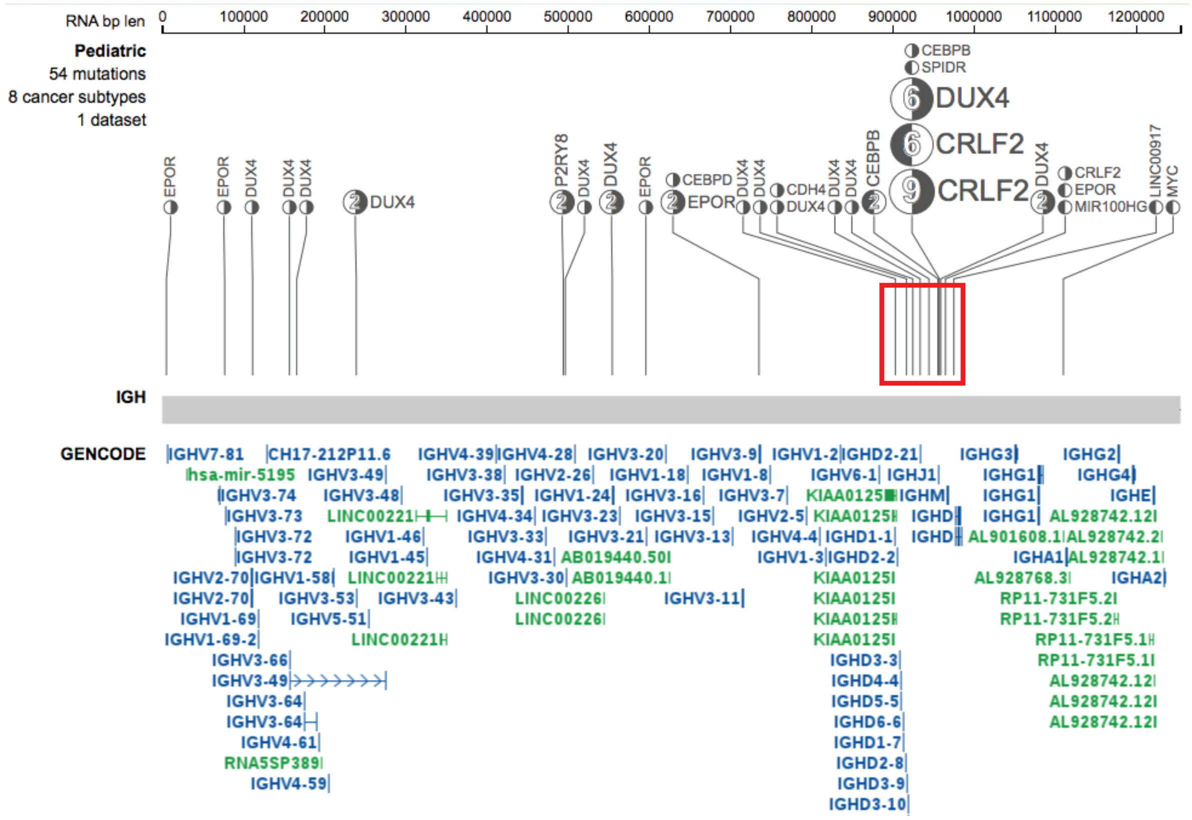

Supplementary Figure 6 The breakpoints of *IGH@* translocations are enriched at *IGHD* and *IGHJ* regions (red box).

54 B-ALL samples with *IGH @* translocations were shown, which were obtained from St. Jude Pediatric Cancer (PeCan) data portal[2].

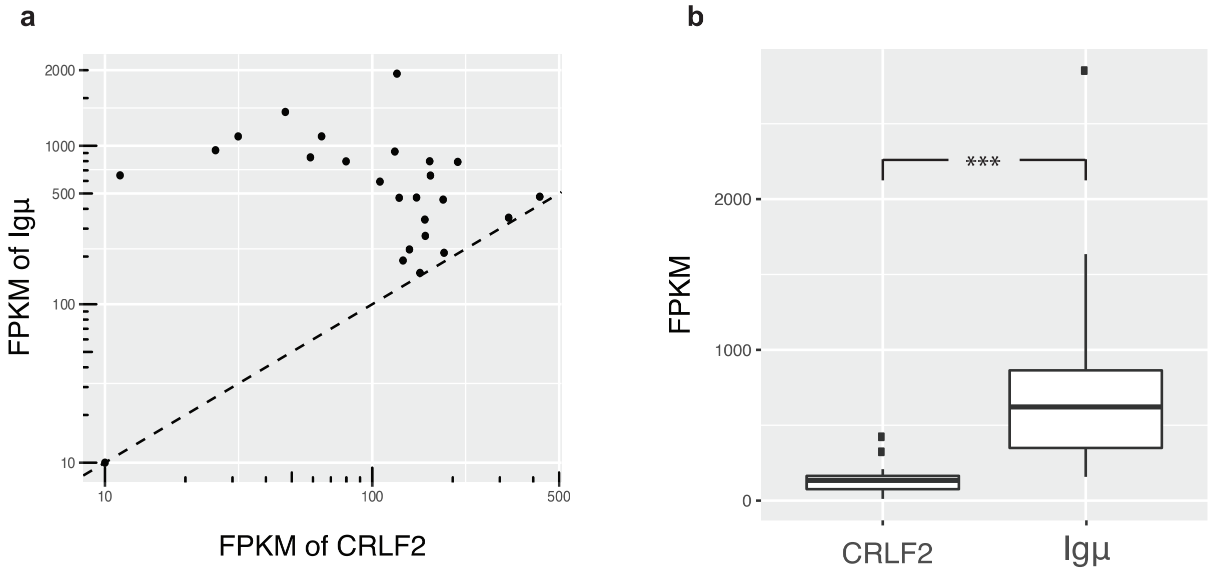

Supplementary Figure 7 The expression of *CRLF2* is much lower than Igµ in B-ALL patients with the *IGH-CRLF2* translocation.

(a) Dot plot of *CRLF2* and Igµ expression in 24 *IGH-CRLF2* B-ALL patients. (b) Box plot of *CRLF2* and Igµ expression in these patients. Median FPKM of *CRLF2* and Igµ in 24 patients are 134.0 and 621.5, respectively. One-tailed, paired sample t-test was performed: *** $p<0.001$. The RNA-seq data are from published paper[3, 4] and available in EGA (EGAS00001000654) and dbGaP (phs000218). Boxes show the first to third quartile with median, whiskers encompass 1.5 times of the interquartile range, and data beyond that threshold indicated as outliers.

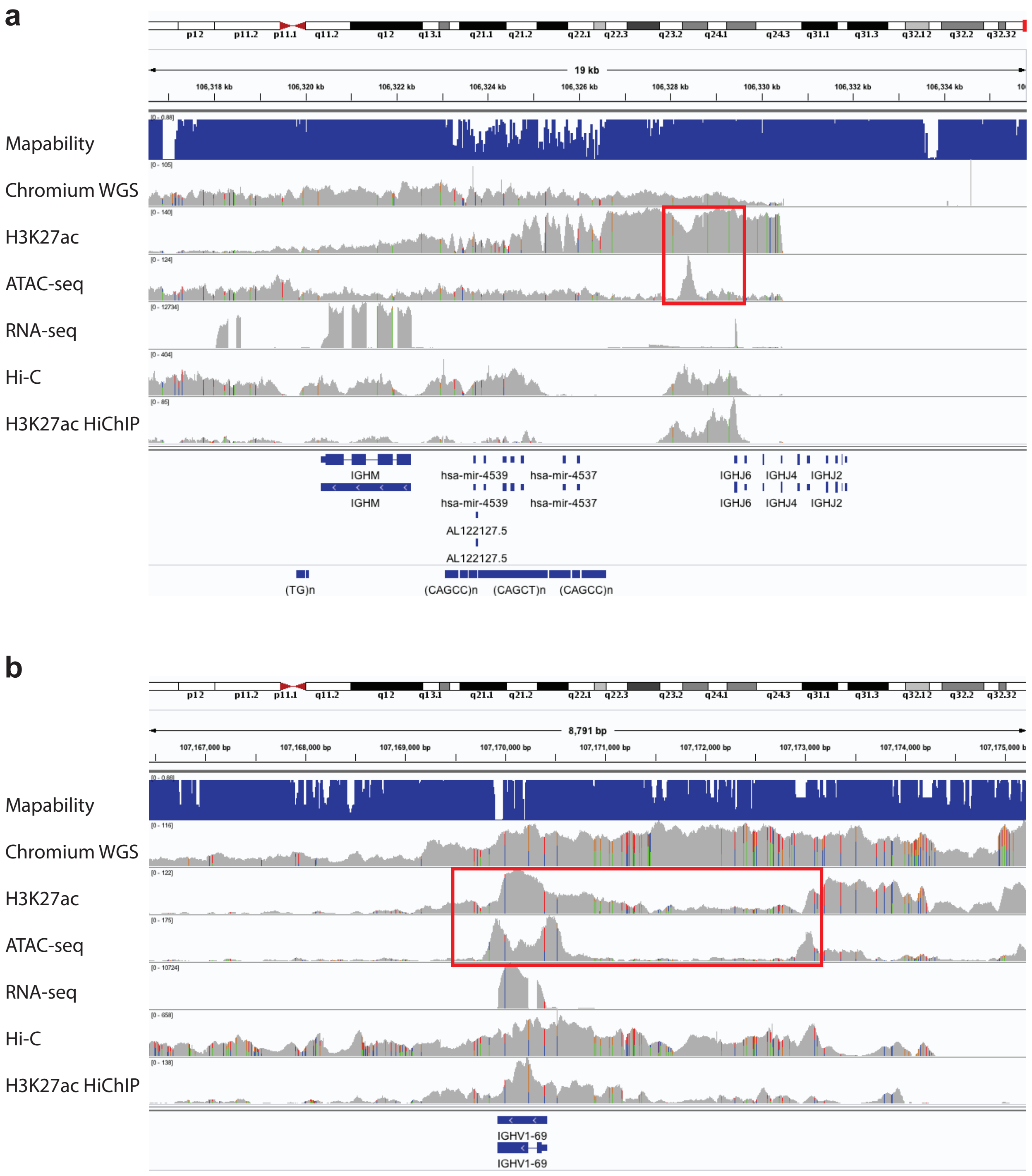
a
19 kb
106,318 kb
106,320 kb
106,322 kb
106,324 kb
106,326 kb
106,328 kb
106,330 kb
106,332 kb
106,334 kb
Mapability
Chromium WGS
H3K27ac
ATAC-seq
RNA-seq
Hi-C
H3K27ac HiChIP
IGHM
IGHM
hsa-mir-4539
hsa-mir-4537
hsa-mir-4539
hsa-mir-4537
AL122127.5
AL122127.5
IGHJ6
IGHJ4
IGHJ2
IGHJ6
IGHJ4
IGHJ2
(TG)n
(CAGCC)n
(CAGCT)n
(CAGCC)n
b
8,791 bp
107,167,000 bp
107,168,000 bp
107,169,000 bp
107,170,000 bp
107,171,000 bp
107,172,000 bp
107,173,000 bp
107,174,000 bp
Mapability
Chromium WGS
H3K27ac
ATAC-seq
RNA-seq
Hi-C
H3K27ac HiChIP
IGHV1-69
IGHV1-69

Supplementary Figure 8 *IGH* enhancer and Igμ promoter regions for allele-specific analysis of enhancer-promoter interaction.

The region enriched with H3K27ac/ATAC-seq signals marked as red rectangle was used as (a) *IGH* intronic enhancer (chr14:106327843-106329701 in hg19) and (b) Igμ promoter (chr14:107169328-107173331 in hg19). The mapability track was downloaded from UCSC genome browser (“wgEncodeDukeMapabilityUniqueness20bp” in hg19).

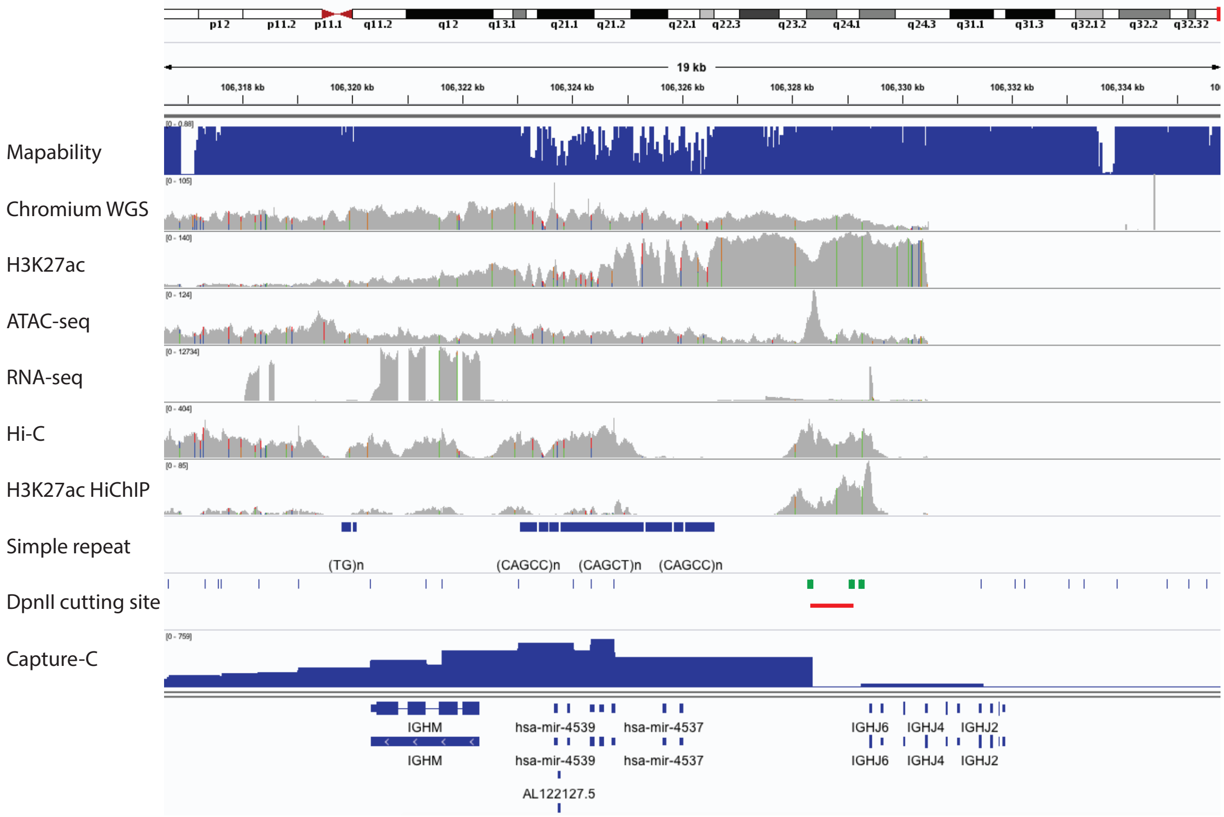

Supplementary Figure 9 Oligo design for the *IGH* intronic enhancer (Eμ) in capture-C.

There were 3 DpnII cutting sites at Eμ region shown as green bars. Three oligos were designed for Eμ within the fragment marked as red bar.

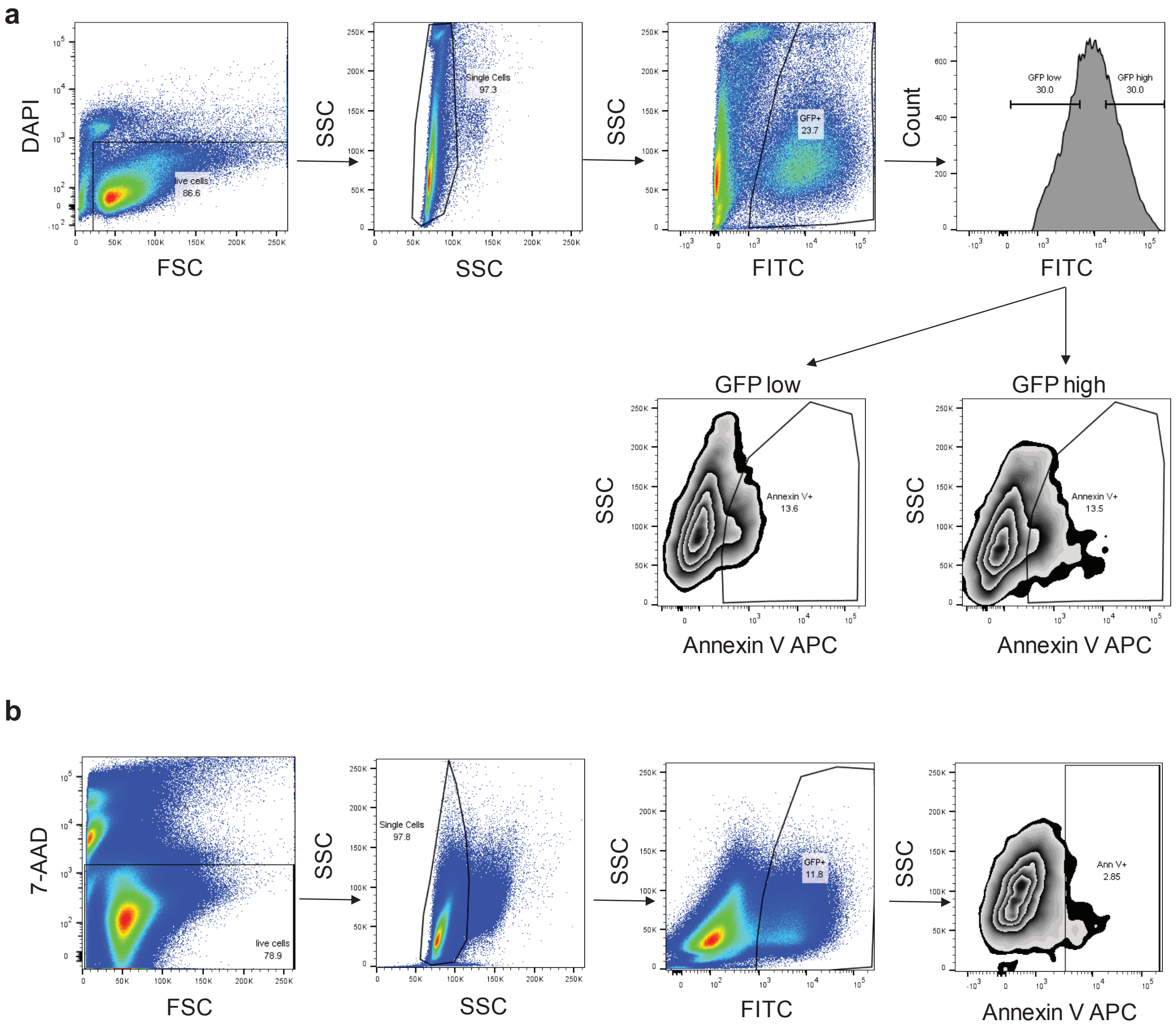

Supplementary Figure 10 Details of the gating strategy used to determine apoptosis for (a) murine bone marrow cells and (b) Nalm6 cells.

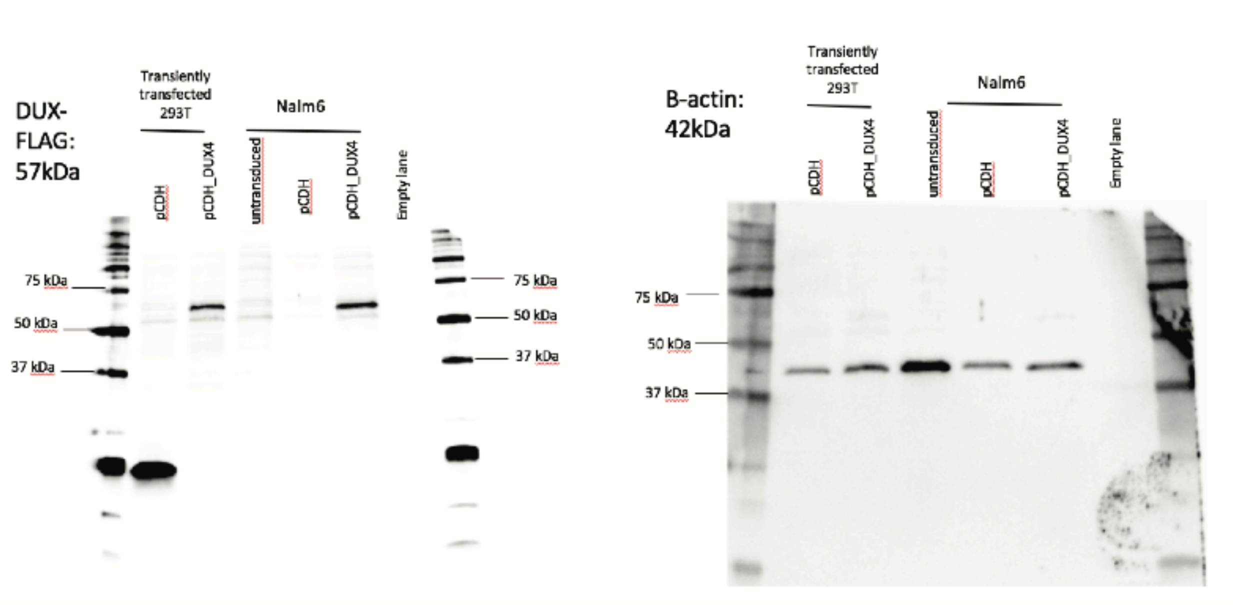

Supplementary Figure 11 Uncropped and unprocessed scans for western blot in Fig. 4c.

Supplementary Table 1: Oligos sequence at *IGH* intronic enhancer for Capture-C

Oligo Coordinate (hg19)	Oligo Sequence
chr14:106328223-106328343	TAAGATTGGAAATGAAATTCAGATAGTTAAAAAAAGCCTTTTCAGTTTCGGTCAGCCTCGCCTTATTTTAGAAACGCAAATTGTCCAGGTGTTGTTTTGCTCAGTAGAGCACTTTCAGATC
chr14:106328340-106328460	GATCTGGGCCTGGGCAAAACCACCTCTTCACAACCAGAAGTGATAAATTTACCAATTGTGTTTTTTTGCTTCCTAAAATAGACTCTCGCGGTGACCTGCTTCCTGCCACCTGCTGTGGGTG
chr14:106328962-106329082	GGATTCACCCACTCCGACAGTTCTCTTTCCAGCCAATAAAGAATTTAAGATGCAGGTTGACACACAGCGCACCTCATAATTCTAAAGAAAATATTTCACGATTCGCTGCTGTGCAGCGATC

Supplementary References

1. Yasuda T*, et al.* Recurrent DUX4 fusions in B cell acute lymphoblastic leukemia of adolescents and young adults. *Nat Genet* **48**, 569-574 (2016).

2. Zhou X*, et al.* Exploring genomic alteration in pediatric cancer using ProteinPaint. *Nat Genet* **48**, 4-6 (2016).

3. Ma X*, et al.* Pan-cancer genome and transcriptome analyses of 1,699 paediatric leukaemias and solid tumours. *Nature* **555**, 371-376 (2018).

4. Roberts KG*, et al.* Targetable kinase-activating lesions in Ph-like acute lymphoblastic leukemia. *N Engl J Med* **371**, 1005-1015 (2014).
