# Supplementary material for: Long-read sequencing unveils IGH-DUX4 translocation into the silenced IGH allele in B-cell acute lymphoblastic leukemia
Source: Nat Commun. 2019 Jun 26;10:2789. doi: 10.1038/s41467-019-10637-8 (PMC6594946; doi:10.1038/s41467-019-10637-8)
Supplement: Supplementary file 3 — Reporting Summary [file 41467_2019_10637_MOESM3_ESM.pdf]

nature research

Corresponding author(s): Jinghui Zhang

Last updated by author(s): 2019-4-3

Reporting Summary

Nature Research wishes to improve the reproducibility of the work that we publish. This form provides structure for consistency and transparency in reporting. For further information on Nature Research policies, see Authors & Referees and the Editorial Policy Checklist.

Statistics

For all statistical analyses, confirm that the following items are present in the figure legend, table legend, main text, or Methods section.

n/a	Confirmed	
☐	☒	The exact sample size (*n*) for each experimental group/condition, given as a discrete number and unit of measurement
☐	☒	A statement on whether measurements were taken from distinct samples or whether the same sample was measured repeatedly
☐	☒	The statistical test(s) used AND whether they are one- or two-sided<br>*Only common tests should be described solely by name; describe more complex techniques in the Methods section.*
☒	☐	A description of all covariates tested
☒	☐	A description of any assumptions or corrections, such as tests of normality and adjustment for multiple comparisons
☐	☒	A full description of the statistical parameters including central tendency (e.g. means) or other basic estimates (e.g. regression coefficient) AND variation (e.g. standard deviation) or associated estimates of uncertainty (e.g. confidence intervals)
☒	☐	For null hypothesis testing, the test statistic (e.g. *F*, *t*, *r*) with confidence intervals, effect sizes, degrees of freedom and *P* value noted<br>*Give P values as exact values whenever suitable.*
☒	☐	For Bayesian analysis, information on the choice of priors and Markov chain Monte Carlo settings
☒	☐	For hierarchical and complex designs, identification of the appropriate level for tests and full reporting of outcomes
☐	☒	Estimates of effect sizes (e.g. Cohen's *d*, Pearson's *r*), indicating how they were calculated

Our web collection on statistics for biologists contains articles on many of the points above.

Software and code

Policy information about availability of computer code

Data collection	RNA-seq data (GSE85632) for human cleavage cells were downloaded from the GEO database by SRA toolkit v2.8.2-1.
Data analysis	STAR v2.5: Aligning RNA-seq/Iso-Seq reads<br>HTseq-count: Calculating FPKM<br>Long Ranger 2.0.0 pipeline: variant calling and phasing from 10x Genomics Chromium WGS<br>Lariat: Aligning 10x Genomics Chromium WGS reads<br>Freebayes v0.9.21: haplotype-based variants calling from 10x Genomics Chromium WGS<br>bcftools v1.1: variants filtering<br>Supernova v1.1.4: de novo DNA assembly from 10x Genomics Chromium WGS<br>cutadapt v1.9: trimming adapter from ATAC-seq reads<br>BWA v0.7.12-r1039: Aligning ATAC-seq/ChIP-seq reads<br>Picard v2.6.0: marking duplicated read from ATAC-seq/ChIP-seq reads<br>samtools v1.2: removing duplicated read from ATAC-seq/ChIP-seq reads<br>SPP: draw cross-correlation plot and calculated relative strand correlation value from ChIP-seq reads<br>bsmap v2.74: Aligning WGBS reads<br>Juicer v1.5: analyzing Hi-C reads<br>HiC-Pro: analyzing HiChIP reads<br>captureC pipeline: analyzing Capture-C reads

For manuscripts utilizing custom algorithms or software that are central to the research but not yet described in published literature, software must be made available to editors/reviewers. We strongly encourage code deposition in a community repository (e.g. GitHub). See the Nature Research guidelines for submitting code & software for further information.

Data

Policy information about availability of data

All manuscripts must include a data availability statement. This statement should provide the following information, where applicable:

- Accession codes, unique identifiers, or web links for publicly available datasets
- A list of figures that have associated raw data
- A description of any restrictions on data availability

10X Chromium WGS, PacBio Iso-Seq data, and bisulfite sequencing data at IGHM region are available in NCBI BioProject PRJNA473990 [https://www.ncbi.nlm.nih.gov/sra/PRJNA473990]. H3K27ac ChIP-seq, Hi-C, HiChIP, Capture-C, and ATAC-seq data are available in the GEO database (GSE115494 [https://www.ncbi.nlm.nih.gov/geo/query/acc.cgi?acc=GSE115494]).

Field-specific reporting

Please select the one below that is the best fit for your research. If you are not sure, read the appropriate sections before making your selection.

☒ Life sciences ☐ Behavioural & social sciences ☐ Ecological, evolutionary & environmental sciences

For a reference copy of the document with all sections, see nature.com/documents/nr-reporting-summary-flat.pdf

Life sciences study design

All studies must disclose on these points even when the disclosure is negative.

Sample size	Previously published B-ALL RNA-seq data from 32 IGH-DUX4 and 24 IGH-CRLF2 cases were used for the analysis.
Data exclusions	No data exclusions
Replication	The number of independent experiments performed for DUX4 toxicity experiment in murine bone marrow cells and Nalm6 cell line is defined in the figure legend. All attempts at replication was successful in this study.
Randomization	No randomization was performed for this study.
Blinding	No blinding was performed for this study.

Reporting for specific materials, systems and methods

We require information from authors about some types of materials, experimental systems and methods used in many studies. Here, indicate whether each material, system or method listed is relevant to your study. If you are not sure if a list item applies to your research, read the appropriate section before selecting a response.

Materials & experimental systems

n/a	Involved in the study	
☐	☒	Antibodies
☐	☒	Eukaryotic cell lines
☒	☐	Palaeontology
☐	☒	Animals and other organisms
☒	☐	Human research participants
☒	☐	Clinical data

Methods

n/a	Involved in the study	
☐	☒	ChIP-seq
☐	☒	Flow cytometry
☒	☐	MRI-based neuroimaging

Antibodies

Antibodies used	Anti-FLAG (Sigma F3165; 10ug/ml), anti-Beta Actin (Abcam ab8227; 1:1000 dilution), anti-mouse IgG superclonal-HRP (Invitrogen A28177; 1:5000 dilution), anti-rabbit-HRP (Abcam ab6721-1; 1:3000 dilution)
Validation	All antibodies were obtained from commercial sources and are certified through the companies standard validation process to ensure quality and reproducibility.

Eukaryotic cell lines

Policy information about cell lines

Cell line source(s)	Nalm6: ATCC, Manassas, VA, USA

Authentication	The DUX4-IgM fusion was verified in Nalm-6 cells by western blot which served to authenticate the cell line.
Mycoplasma contamination	Cell line was not tested for mycoplasma contamination since purchase from ATCC.
Commonly misidentified lines (See ICLAC register)	Nalm6 is not listed in commonly misidentified cell lines in ICLAC based on the latest version (Version 9, released 14 October 2018).

Animals and other organisms

Policy information about studies involving animals; ARRIVE guidelines recommended for reporting animal research

Laboratory animals	C57/BL6 4-6 weeks of age. Both male and female bone marrow was utilized.
Wild animals	No wild animals
Field-collected samples	No field collected samples
Ethics oversight	All animal studies were maintained and treated in accordance with guidelines approved by the Institutional Animal Care and Use Committee (IACUC) at St. Jude Children's research hospital.

Note that full information on the approval of the study protocol must also be provided in the manuscript.

ChIP-seq

Data deposition

☒ Confirm that both raw and final processed data have been deposited in a public database such as GEO.

☒ Confirm that you have deposited or provided access to graph files (e.g. BED files) for the called peaks.

Data access links<br>*May remain private before publication.*	H3K27ac ChIP-seq data is available in the GEO database (GSE115494 [https://www.ncbi.nlm.nih.gov/geo/query/acc.cgi?acc=GSE115494]).
Files in database submission	GSM3210231 H3K27ac ChIP-seq<br>GSM3210233 Input ChIP-seq
Genome browser session (e.g. UCSC)	None

Methodology

Replicates	One replicate from Active Motif. Because our purpose is to evaluate allele-specific event for H3K27ac instead of peak calling, one replicate is performed.
Sequencing depth	For the H3K27ac ChIP-seq, a frozen cell pellet containing 10 million cells was sent to Active Motif for ChIP and library preparation. The sample was divided into an aliquot for ChIP using an antibody to H3K27ac (Active Motif) and an input control. Single-end sequencing was performed using an Illumina NextSeq 500 generating 76 cycles for each sequencing read. Total 96,160,526 reads. 41,242,667 reads with MAPQ>=1; read length single-end 76bp;
Antibodies	H3K27ac antibody #33201 (Active Motif AM cat# 39133)
Peak calling parameters	No peak calling in this study
Data quality	For the H3K27ac ChIP-seq, we followed ENCODE guideline41 for quality control. Briefly, we used SPP to draw cross-correlation plot and calculated relative strand correlation value (RSC). We then estimate the fragment size from corss-coorelation plot and extended each read to the estimated fragment size to generate bigwig files (normalized to 15 million unique mapped reads). Observed clear peak shape along with RSC > 1 and ~40M (ENCODE criterion 10M) unique mapped reads, we conclude our IP sample for H3K27ac is good.
Software	For the H3K27ac ChIP-seq, the reads were mapped to human genome hg19 (GRCh37-lite) by BWA (version 0.7.12-r1039, default parameter)34, duplicated reads were then marked with Picard(version 2.6.0-SNAPSHOT)35 and only non-duplicated reads have been kept by samtools (parameter "-q 1 -F 1024" version 1.2).

Flow Cytometry

Plots

Confirm that:

☒ The axis labels state the marker and fluorochrome used (e.g. CD4-FITC).

☒ The axis scales are clearly visible. Include numbers along axes only for bottom left plot of group (a 'group' is an analysis of identical markers).

☒ All plots are contour plots with outliers or pseudocolor plots.

☒ A numerical value for number of cells or percentage (with statistics) is provided.

Methodology

Sample preparation	Cells were washed with Annexin V staining buffer and stained with annexin V conjugated to APC (Tonbo Biosciences, San Diego, CA, USA) for 15 min at 25 °C, washed and resuspended in 4',6-diamidino-2-phenylindole containing solution (1 µg/ml DAPI) or 7-Aminoactinomycin A solution (7-AAD)
Instrument	FACS LSR II D (BD Biosciences, San Jose, CA, USA)
Software	FlowJo (FlowJo LLC, Ashland, Oregon, USA)
Cell population abundance	Following transduction of Nalm6, cells were flow sorted to purity for western blot analysis (Figure 4c). Sorting gates were selected such that the GFP positive sorted population was >99% pure. Cells were sorted from each experiment and pooled for Western blotting. Each experiment yielded approximately 100,000 purified cells.
Gating strategy	In all experiments, live cells were gated on as defined by DAPI or 7-AAD exclusion. GFP positive gate was set based on the GFP negative parental cell line or GFP negative bone marrow, and chosen to reflect less than 0.05% of events. Gating strategy for bone marrow analysis which defined GFP high and low populations is shown in Figure 4a. Annexin V positive gate was established by unstained controls as specified in the technical data sheet by the manufacturer and chosen to reflect less than 0.2% of events.

☒ Tick this box to confirm that a figure exemplifying the gating strategy is provided in the Supplementary Information.
